# Non-Noble FeCrO*_x_* Bimetallic Nanoparticles for Efficient NH_3_ Decomposition

**DOI:** 10.3390/nano13071280

**Published:** 2023-04-05

**Authors:** Meng Du, Lingling Guo, Hongju Ren, Xin Tao, Yunan Li, Bing Nan, Rui Si, Chongqi Chen, Lina Li

**Affiliations:** 1 Shanghai Institute of Applied Physics, Chinese Academy of Sciences, Shanghai 201204, China; dum@sari.ac.cn (M.D.);; 2 Shanghai Synchrotron Radiation Facility, Shanghai Advanced Research Institute, Chinese Academy of Sciences, Shanghai 201210, China; 3 University of Chinese Academy of Sciences, Beijing 100049, China; 4 National Engineering Research Center of Chemical Fertilizer Catalyst, Fuzhou University, Gongye Road 523, Fuzhou 350002, China

**Keywords:** ammonia decomposition, FeCr bimetallic oxide, CO*_x_*-free hydrogen, XAFS

## Abstract

Ammonia has the advantages of being easy to liquefy, easy to store, and having a high hydrogen content of 17.3 wt%, which can be produced without CO*_x_* through an ammonia decomposition using an appropriate catalyst. In this paper, a series of FeCr bimetallic oxide nanocatalysts with a uniform morphology and regulated composition were synthesized by the urea two-step hydrolysis method, which exhibited the high-performance decomposition of ammonia. The effects of different FeCr metal ratios on the catalyst particle size, morphology, and crystal phase were investigated. The Fe_0.75_Cr_0.25_ sample exhibited the highest catalytic activity, with an ammonia conversion of nearly 100% at 650 °C. The dual metal catalysts clearly outperformed the single metal samples in terms of their catalytic performance. Besides XRD, XPS, and SEM being used as the means of the conventional characterization, the local structural changes of the FeCr metal oxide catalysts in the catalytic ammonia decomposition were investigated by XAFS. It was determined that the Fe metal and FeN*_x_* of the *bcc* structure were the active species of the ammonia-decomposing catalyst. The addition of Cr successfully prevented the Fe from sintering at high temperatures, which is more favorable for the formation of stable metal nitrides, promoting the continuous decomposition of ammonia and improving the decomposition activity of the ammonia. This work reveals the internal relationship between the phase and structural changes and their catalytic activity, identifies the active catalytic phase, thus guiding the design and synthesis of catalysts for ammonia decomposition, and excavates the application value of transition-metal-based nanocomposites in industrial catalysis.

## 1. Introduction

The shortage of fossil fuels is driving scientists to actively pursue alternative energy sources. For many years, hydrogen energy (H_2_) has been regarded as a clean and environmentally friendly source, but the complicated storage and release of hydrogen are limiting factors for its wide application. Ammonia (NH_3_) is considered to be a desirable hydrogen carrier due to its unique properties, such as a high H_2_ storage capacity, of approximately 17 wt%, and a high energy density, relative to that of3 conventional carbon-containing materials. Despite the fact that ammonia is a toxic compound that can cause environmental pollution, NH_3_ can provide CO*_x_*-free hydrogen (H_2_) through the decomposition of ammonia molecules, which is one of the most promising technologies in the hydrogen energy economy [1,2,3], facilitating its practical application in proton-exchange membrane fuel cells (PEMFC) [4]. Furthermore, NH_3_ can be liquefied under mild conditions, making its transportation and storage more convenient. The catalytic production of hydrogen from NH_3_ decomposition is therefore of both fundamental and practical importance. As a result, the catalytic performance and surface chemistry of NH_3_ decomposition have received considerable attention [5,6]. Ammonia decomposition is the process of heat absorption and volume increase, benefiting from high temperatures and low pressures.. The high activation energy of ammonia prevents it from decomposing, even at temperatures of up to 600 °C. The application of suitable catalysts can reduce its activation energy, allowing the reaction to proceed more rapidly. Until now, supported catalysts, including noble metals (Ru, Rh, Ir, and Pt) and non-noble metals (Fe, Co, and Ni), have been developed for ammonia decomposition to produce hydrogen, among which, Ru catalysts have demonstrated excellent catalytic activity [2,7,8,9]. 

However, the widespread application of these active Ru catalysts was constrained by their high cost and the demand for corrosion equipment. Therefore, it is urgently necessary to develop inexpensive non-noble metal catalysts for the hydrogen generation from NH_3_ decomposition. Although non-noble metal catalysts show good catalytic activity for ammonia decomposition, they generally require higher operating temperatures than Ru- based materials. The catalytic properties of Fe [10,11,12,13,14,15,16], Co [17,18,19,20], and Ni [21,22,23,24,25,26] catalysts for ammonia decomposition have been extensively explored over the past few decades. Wang et al. [27] studied the synergistic effect of plasma and Fe, Co, and Ni catalysts, and discovered that the ammonia decomposition activity was increased at least fivefold when compared to the catalyst without plasma. Duan et al. [28] found that the adsorption energy of NH_3_ on Co and Ni was lower than that on Fe, and that Fe had the highest activation energy according to a DFT theoretical study. Compared to Co-based and Ni-based catalysts, Fe-based catalysts usually exhibited the lowest activity, which was also consistent with the experimental results. Although the Fe-based catalysts exhibited a relatively lower activity and stability than the commonly used Ru- and Ni-based catalysts, their low cost and widespread availability make it necessary to develop highly efficient Fe-based catalysts. The Fe-based catalysts are easy to sinter at high temperatures and can be nitrided with NH_3_ to form nitrogen compounds. Somorjai et al. [29,30] studied the Fe single crystals in the ammonia synthesis reaction using field ion microscopy (FIM). The activity of the different crystal planes of α-Fe in the ammonia synthesis was measured at a pressure of 2.0 MPa. The relative activity ratio of the Fe (111), Fe (100), and Fe (110) was 418:25:1. This is mainly due to the presence of more C7 sites (the “site” or “center” surrounded by seven atoms) on the Fe (111) crystal plane, which is known to be highly active in ammonia synthesis. It is worth noting that the ammonia decomposition and ammonia synthesis reaction are reversible reactions, and asking if the ammonia decomposition reaction has similar properties to the ammonia synthesis. Therefore, it is necessary to explore whether the ammonia decomposition reaction with an Fe-based catalyst is, likewise, a structurally sensitive reaction. The lower activity of Fe-based catalysts compared to Ru-based catalysts can be explained by the higher bond enthalpy of Fe-N bonds compared to that of Ru-N, which will result in the formation of surface nitride, slowing down the reaction rate and eventually inactivating the catalyst poisoning. Although iron typically forms stable nitrides and industrial nitrides occurs at 600 °C, it has also been reported to occur at lower temperatures (300 °C) [2]. This inactivation process is reversible at high reaction temperatures, along with the desorption of nitride, but this is usually accompanied by the sintering of iron, resulting in the irreversible passivation of the iron catalyst [10]. However, a major barrier for most supported non-noble metal catalysts has not been overcome, namely, the low ammonia decomposition catalytic activity that is caused by the low loading of active components or the high loading of active species that are seriously sintered at high reaction temperatures. The FeCrO*_x_* metal material has been widely studied by geologists due to its abundant mineral resources and distinctive spinel structure. This catalyst has also been investigated in a high-temperature water–gas shift reaction [31], with remarkable catalytic activity at 350–450 °C. As Fe is easily burned into blocks at high temperatures, it is possible to improve the activity and stability of an Fe-based catalyst by introducing a Cr element to effectively prevent its sintering [32,33,34]. Li and Zhu et al. [35] used the thermal decomposition method to successfully synthesize a mesoporous Cr_2_O_3_ catalyst for ammonia decomposition by adding a CTAB surfactant. The catalytic activity of the Cr_2_O_3_ catalyst for ammonia decomposition was structurally sensitive and closely related to its particle size, which improved with an increase in the particle size. The XPS results indicated that N atoms were introduced into the Cr_2_O_3_ lattice and interstitial CrN*_x_*O*_y_* compounds were generated during the ammonia decomposition reaction, resulting in the formation of strong Cr-N bonds and the hindering of the N reassociation on the Cr_2_O_3_ surface. Thus, it is of academic and practical significance to design the synthesis of new non-noble metal catalysts with a high content of active species, anti-sintering properties, and an outstanding catalytic performance within an ammonia decomposition reaction.

On the other hand, it is crucial to identify the catalytically active species within the NH_3_ decomposition reaction by using appropriate characterization techniques. In the ammonia decomposition reaction, the inference of the active structure of the catalyst is also controversial. Traditional characterization methods are usually used to characterize the phase and surface structure of the catalysts, but the obtained information is rather limited for understanding the structure–activity relationship of the catalytic reaction. Therefore, on the basis of XRD, TEM, and other conventional characterization methods, we attempt to use XAFS to explore the structure–activity relationship of FeCr metal oxides in the catalytic decomposition of ammonia.

In this work, we synthesized a series of FeCr bimetal oxide nanocatalysts with varying metal ratios, and evaluated the modulation effect of these different FeCr metal ratios on the particle size, morphology, and crystal phase of the catalyst. With the help of XRD, XAFS, and other characterization methods, the active species of the reaction was identified. The controlled synthesis of innovative non-noble metal catalysts with outstanding catalytic properties for NH_3_ decomposition is of both academic and practical interest for heterogeneous catalysis.

## 2. Experimental

### 2.1. Catalyst Synthesis

All the chemicals used in this work were of an analytical grade and were purchased from Sinopharm Chemical Reagent Co., Ltd., without any further purification.

The Fe*_x_*Cr*_y_* catalysts that were used in this study, labelled as Fe, Fe_0.75_Cr_0.25_, Fe_0.5_Cr_0.5_, Fe_0.25_Cr_0.75_, and Cr, were prepared by controlling the molar ratios of Fe:Cr as 1:0, 3:1, 1:1, 1:3, and 0:1, respectively, and using two steps of the urea hydrolysis assisted co-precipitation method. The calculated amounts of the iron nitrate (Sigma Aldrich, (Shanghai, China), 99.999% trace metals basis) and chromium nitrate (Sigma Aldrich, (Shanghai, China), 99.99% trace metals basis) precursors and the urea (1.5 mol/L) were mixed and dissolved in deionized water. The resulting mixtures were aged for 1 h and further transferred to 100 mL Teflon-lined stainless steel autoclaves, where they were crystallized at 80 °C for 6 h, and then heated to 180 °C for 24 h. After they were cooled to room temperature, the precipitates were collected by centrifuging and washing with deionized water. Finally, the products were oven-dried overnight at 70 °C and calcined at 400 °C for 4 h.

### 2.2. Characterizations

The powder X-ray diffraction (XRD) patterns were recorded on a Burker D8 Advance diffractometer (40 kV, 40 mA) with a scanning rate of 4° min^−1^, using Cu *K*_α_ radiation (*λ* = 1.5406 Å). The corresponding XRD patterns were collected from 10 to 90°, with a step of 0.02°. The 2*θ* angles were calibrated with a μm-scale Alumina disc. After grinding, the powder catalyst was placed inside a quartz–glass sample holder for each test. With the software “LAPOD” for the least-squares refinement, the cell dimensions from the powder data were obtained with Cohen’s method [36,37].

The nitrogen adsorption–desorption measurements were performed on an ASAP2020-HD88 analyzer (Micromeritics Co. Ltd., Norcross, GA, USA) at 77 K. The samples were degassed at 250 °C for 4 h under vacuum. The BET specific surface areas were calculated from the data in the relative pressure range, between 0.06 and 0.30.

The X-ray photoelectron spectroscopy (XPS) analysis was performed on an Axis Ultra XPS spectrometer (Kratos Analytical Ltd, Manchester, UK) with a 225 W Al-*K_α_* radiation source (*E_b_* = 1486.7 eV), which operated at 15 kV and 15 mA. The C 1s line at 284.8 eV was used to calibrate the binding energies. The X-ray photoelectron spectroscopy (XPS) for the used samples was performed by PHI 5000 VersaProbe III, using a monochromatic Al Kα X-ray source with a beam size of 100 μm × 1400 μm. The charge compensation was achieved with the dual beam charge neutralization, and the binding energy was corrected by setting the binding energy of the hydrocarbon C 1s feature to 284.8 eV. The curve fitting was performed with PHI MultiPak software, and Gaussian–Lorentz functions and Shirley background were used.

The scanning electron microscopy (SEM) experiments were carried out on an LEO 1530VP (Zeiss, Jena, Germany) scanning electron microscope with a thermal field emitting electron gun. Its resolutions were a high vacuum mode of 1.0 nm @ 20 kV, WD = 2 m, a magnification of 20x–900,000x, a sample current of 4 pA–10 nA, and an acceleration voltage of 0.1 kV–30 kV.

The X-ray absorption fine structure (XAFS) spectra at the Fe *K*-edge (*E*_0_ = 7112 eV) and Cr *K*-edge (*E*_0_ = 5989 eV) were performed at the BL14W1 beam line of the Shanghai Synchrotron Radiation Facility (SSRF), and operated at 3.5 GeV under “top-up” mode with a constant current of 240 mA. The XAFS data were recorded under transmission mode. The energy was calibrated according to the absorption edge of the pure Fe and Cr foil. Athena and Artemis codes were used to extract the data and fit the profiles. For the X-ray absorption near edge structure (XANES) part, the experimental absorption coefficients, as functions of energies *μ*(*E*), were processed by a background subtraction and normalization procedures, and reported as “normalized absorption”. For the extended X-ray absorption fine structure (EXAFS) part, the Fourier transformed (FT) data in the *R* space were analyzed by applying a 1st shell approximation or multiple Fe compound models for the Fe-O, Fe-N, or Fe-Fe shells, respectively. The passive electron factors, *S*_0_^2^, were determined by fitting the experimental Fe foil data and fixing the Fe-Fe coordination number (*CN*) to be 8+6, and they were then fixed for a further analysis of the measured samples. For Cr, the Fourier transform (FT) in the R-space was analyzed by applying the first shell approximation of the Cr_2_O_3_ and CrN models to Cr-O and Cr-N, respectively. Similarly, the amp was obtained by fixing the coordination number (*CN*) of Cr-Cr as 6, according to the data of the Cr foil, and then the amp was fixed to further analyze the measured samples. The parameters describing the electronic properties (e.g., correction to the photoelectron energy origin, *E*_0_) and local structure environment, including *CN*, bond distance (*R*), and the Debye Waller (*D.W.*) factor around the absorbing atoms, were allowed to vary during the fitting process. The fitted ranges for *k* were selected to be *k* = 2.4–11.1 Å^−1^ and 2.8–12.5 Å^−1^ (*k*^3^ weighted) for the Fe and Cr samples. The Fourier transformed (FT) data in the *R* space were analyzed by selecting *R* = 1.1–3.2 (Fe-Fe) and 1.7–2.7 (Cr-Cr) Å (*k*^3^ weighted), respectively.

### 2.3. Catalytic Tests

Ammonia decomposition was examined in a fixed-bed continuous-flow quartz reactor at atmospheric pressure. Catalytic tests on the catalysts (50 mg) were carried out using pure NH_3_ as a reaction gas, at a flow rate of 19 mL min^−1^, corresponding to a space velocity of 22,000 cm^3^ $g_{cat}^{-1}$ h^−1^. The ammonia conversion of each catalyst was performed in two rounds of identical tests. The first heating process was the pre-activation of the catalyst, then the temperature was lowered to room temperature under the conditions of the NH_3_ flow, and the second round was the same temperature procedure as the first run. During each round test, the catalyst was heated to pre-programmed temperature values by ramping the temperature up from 400 to 650 °C at steps of 50 °C, and each temperature point was maintained for 1 h until a steady state was reached. The outlet gas analysis was performed with an online gas chromatograph (Ouhua GC 9160) that was equipped with a TCD and a Poropak Q column, using H_2_ as the carrier gas. The data were recorded when the signal was stabilized, and the NH_3_ conversion was calculated by normalizing the N atoms. To facilitate a differentiation from the pre-synthesized catalysts, the catalysts that recovered after the ammonia decomposition reaction were successively denoted as Fe-used, Fe_0.75_Cr_0.25_-used, Fe_0.5_Cr_0.5_-used, Fe_0.25_Cr_0.75_-used, and Cr-used.

To evaluate the catalytic stability of the FeCrO*_x_* sample at 600 °C, 66 mg of the catalyst and a space velocity of 22,000 cm^3^  $g_{\mathrm{cat}}^{-1}$ h^−1^ were chosen. The reaction was maintained for 48 h and the NH_3_ conversion was recorded continuously. An online gas chromatography (GC9790 Plus, FULI Instruments, Zhejiang, China) that was equipped with a TCD was applied to analyze the gas composition, using H_2_ as the carrier. The NH_3_ conversion was calculated with Equation (1) and the H_2_ formation rate was calculated with Equation (2). The apparent activation energy (Ea) for the ammonia decomposition was determined at an equal NH_3_ conversion below 20% (to eliminate the mass and heat transfer effects) by tuning the temperature (T), which can be calculated with Equation (3), where R is the universal gas constant and A is a preexponential factor.

(1) $$\eta_{{\mathrm{NH}}_{3}}=\frac{X_{{\mathrm{NH}}_{3},\mathrm{in}}-X_{{\mathrm{NH}}_{3},\mathrm{out}}\;}{X_{{\mathrm{NH}}_{3},\mathrm{in}}}\times 100\%$$

(2) $$H_{2}\;\mathrm{formation}\;\mathrm{rate}\;\left({\mathrm{mmol}\;g}_{\mathrm{cat}}^{-1}{\;\min}^{-1}\right)=\frac{\frac{50}{22.4}\times \eta_{{\mathrm{NH}}_{3}}\times 1.5}{m_{\mathrm{cat}}}$$

(3) $$\ln\left(H_{2}\;\mathrm{formation}\;\mathrm{rate}\right)=-\frac{E\alpha }{R}\times \frac{1}{T}+\mathrm{lnA}$$

The temperature-programmed reduction with hydrogen (H_2_-TPR) was performed in a Builder PCSA-1000 instrument that was equipped with a thermal conductivity detector (TCD) to detect the H_2_ consumption. The sieved catalysts (30 mg, 40–60 mesh) were pretreated in pure O_2_ at 300 °C for 30 min before test. The reduction process was carried out in a gas mixture of 5% H_2_/Ar (30 mL/min) from room temperature to 800 °C (5 °C min^−1^).

## 3. Result and Discussion

### 3.1. Catalyst Structure and Ammonia Decomposition Reactivity

**Figure 1:**
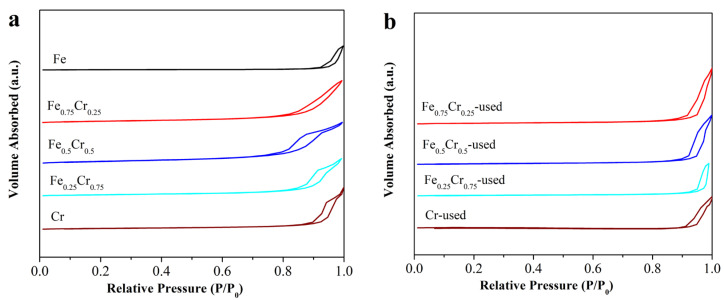
N_2_ adsorption–desorption isotherms of (**a**) fresh, and (**b**) used FeCrO*_x_* catalysts.

The fresh FeCrO*_x_* catalysts were characterized by XPS to identify the surface atomic ratios between the Fe and Cr. As shown in Table 1, the ratios of the surface iron and chromium concentrations are basically compatible with the feeding mole ratios, demonstrating that our two-step urea hydrolysis method could effectively synthesize a sample with the expected percentage of the metal elements. A N_2_ adsorption–desorption experiment was used to examine the structure and mesoporous nature of the fresh and used FeCrO*_x_* catalysts. The adsorption–desorption isotherms of several FeCrO*_x_* catalysts are depicted in Figure 1. The related Brunauer–Emmett–Teller (BET) surface areas are summarized in Table 1. The specific surface areas of the fresh Fe, Fe_0.75_Cr_0.25,_ Fe_0.5_Cr_0.5,_ Fe_0.25_Cr_0.75_, and Cr were 13 m^2^/g, 62 m^2^/g, 84 m^2^/g, 51 m^2^/g, and 39 m^2^/g, respectively. The nitrogen adsorption and desorption isotherms of all the catalysts displayed type V curves, indicating that the interaction between the adsorbate and adsorbent is weak, and that the appearance of the hysteresis loop is induced by the pores between the particles (Figure 1). After the ammonia decomposition reaction, the isotherms of the nitrogen absorption and desorption of each catalyst were similar to those of the samples before the reaction, indicating that the structures of the catalysts were almost preserved. However, the specific surface areas of the samples were reduced after the reaction (Table 1), which was mainly caused by the sintering and agglomeration of the active species during the high-temperature reaction. It is noteworthy that the specific surface area of the pure Fe sample after the reaction was too small to be measured. This can also be confirmed from the SEM results.

**Figure 2:**
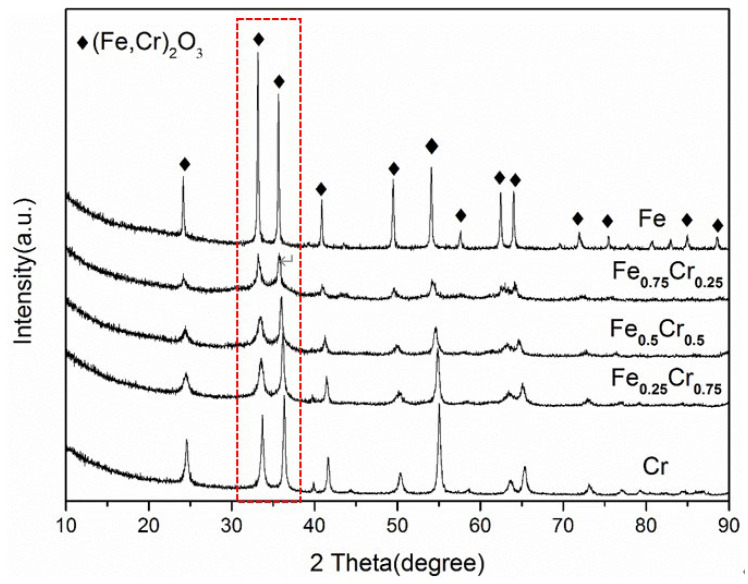
XRD patterns of fresh FeCrO*_x_* catalysts.

The powder XRD data of the fresh samples are shown in Figure 2. The diffraction peaks of the (Fe,Cr)_2_O_3_ phase (JCPDS No. 34-0412) were clearly observed for all these fresh samples, and with the rise in the Cr concentration, the diffraction peaks shifted regularly to a higher angle. Because the ionic radiuses of the Fe^3+^ and Cr^3+^ are very close (Fe^3+^ is 0.645 Å and Cr^3+^ is 0.615 Å) [38], the widening of the XRD peak is mainly related to the inhomogeneity of the cell size, resulting from the spatial changes in the amount and distribution of the Cr^3+^ and Fe^3+^ cations and vacancies [39], and all five samples belong to the same *bcc* rhomboid lattice structure. Furthermore, we calculated the lattice constants based on the XRD patterns of the (Fe,Cr)_2_O_3_ phase and crystal grain sizes, using the Scherrer formula at 33°, which was assigned to the (104) crystal plane of (Fe,Cr)_2_O_3_ for the fresh samples (Table 1). It can be seen from Table 1 that the lattice constants (*a* and *c*) of these samples decrease gradually with an increase in the Cr ion content, and the calculated crystallite size of the (Fe,Cr)_2_O_3_ phase in the pure Fe sample is much larger than that of the samples that contained a Cr element, corresponding to the obviously widened diffraction peaks and weakened strength, as well as the pure Cr sample. From the above results, it can be clearly concluded that the addition of Cr can effectively reduce the grain size of the catalyst samples, which may have a positive effect on the catalytic ammonia decomposition process, such as an increase in the contact area during the reaction.

**Figure 3:**
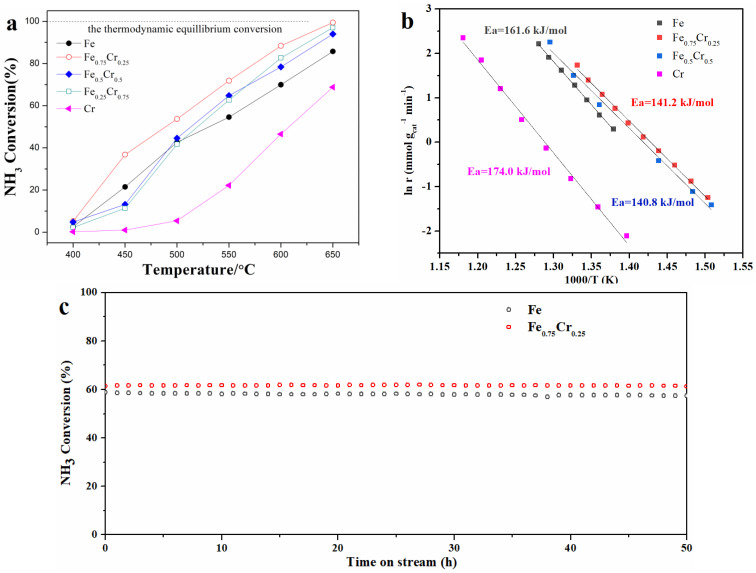
(**a**) NH_3_ conversion as a function of reaction temperature during NH_3_ decomposition reaction with the FeCrO*_x_* catalysts, and the dotted line is the data of the thermodynamic equilibrium [43]. (**b**) Arrhenius plots of NH_3_ conversion over FeCrO*_x_* catalysts, and (**c**) stability tests of Fe and Fe_0.75_Cr_0.25_ catalysts. All catalysts’ performances were tested with a gas hourly space velocity (GHSV) of 22,000 cm^3^ $g_{\mathrm{cat}}^{-1}$ h^−1^.

The catalytic activities of the FeCrO*_x_* catalysts in the ammonia decomposition were examined in two runs, with the same space velocity of 22,000 cm^3^ $g_{\mathrm{cat}}^{-1}$ h^−1^ of the pure NH_3_ flow. During the catalytic reaction process, each chosen temperature point was maintained for 1 h, and the final reported catalyst activity curves were obtained by using the last data that were collected from the GC. The first heating process mainly involved sample activation. In the second heating process, the ammonia conversions of the FeCrO*_x_* catalysts in the temperature range of 400 °C to 650 °C are shown in Figure 3a. The Fe_0.75_Cr_0.25_ sample exhibited the highest catalytic activity in the continuous tests, with an ammonia conversion of nearly 40% at 450 °C, exceeding that of the other catalysts. Subsequently, the ammonia decomposition rate increased approximately linearly from 500 °C to 650 °C, reaching almost 100% at 650 °C. The ammonia conversions were slightly lower for the Fe_0.5_Cr_0.5_ and Fe_0.25_Cr_0.75_ catalysts than for the Fe_0.75_Cr_0.25_ sample at low temperatures (400–550 °C), but tended to be quite close as the reaction temperature increased, demonstrating that the Fe-Cr composite catalysts could effectively promote ammonia decomposition at a high reaction temperature. When compared to the other Cr-doping catalysts, the pure Fe sample, which was composed of Fe_2_O_3_ species, exhibited a higher activity below 500 °C, but lower catalytic activities as the reaction temperature increased, mainly because of the sintering effect of such iron catalysts [10]. The NH_3_ conversion at 650 °C was only about 84%, which is significantly lower than that of the Fe_0.75_Cr_0.25_ and other samples. On the other hand, the ammonia conversion of the pure Cr sample (mainly Cr_2_O_3_) was lower than that of all the Fe-Cr composite catalysts, and the results were consistent with previously reported Fe- and Cr-based catalysts used within the ammonia decomposition reaction [35,40,41,42]. A comparison of the ammonia decomposition activities of various Fe-based catalysts, including data from the literature, is summarized in Table 2. The catalytic performance of the Fe_0.75_Cr_0.25_ catalyst for the ammonia decomposition was comparable to those of non-noble Fe-based catalysts that have been reported in recent years. Figure 3b shows the Arrhenius-type plots of the NH_3_ decomposition over the Fe, Fe_0.75_Cr_0.25_, Fe_0.5_Cr_0.5_, and Cr samples, and the corresponding apparent activation energies, which are 161.6 141.2, 140.8, and 174 kJ mol^−1^, respectively. These values are consistent with the catalytic activities. The reaction is more unfavorable and results in a decrease in activity with a higher activation energy. In addition, the similar activation energies of Fe_0.75_Cr_0.25_ and Fe_0.5_Cr_0.5_ (~140 kJ mol^−1^) suggest that they have similar active sites and follow similar ammonia decomposition reaction pathways. The Fe_0.75_Cr_0.25_ and Fe catalysts underwent a long-term stability test at 600 °C, with a GHSV of 22,000 cm^3^ $g_{\mathrm{cat}}^{-1}$ h^−1^. It can be observed from Figure 3c that the NH_3_ conversion had almost no decrease and was maintained at 60%, even after 50 h of measurement, further highlighting the excellent high-temperature stability of FeCrO*_x_* catalysts.

**Table 2:** Comparison of ammonia conversion with different Fe-based catalysts.

Catalyst	Temperature	GHSV (NH_3_ cm^3^ $g_{\mathrm{cat}}^{-1}$ h^−1^)	Conversion (%)	Reference
**Fe_0.75_Cr_0.25_**	600	22,000	88.4	This work
**Fe_0.75_Cr_0.25_**	650	22,000	99.5	This work
**Fe-Al_2_O_3_**	600	36,000	86	[40]
**Fe/SiO_2_**	600	15,000	65	[44]
**Fe/CMK-5**	600	7500	96	[45]
**Fe-CNTs**	700	5000	75	[46]
**CoFe_5_/CNTs**	600	36,000	50	[47]
**Fe/SiO_2_-Cs**	600	30,000	90	[48]
**Fe-Mo**	550	46,000	16	[49]
**Fe-Co**	550	6000	77	[50]
**Fe-Mg**	550	6000	86	[50]

### 3.2. The Morphology and Crystal Structure of Used FeCrO_x_ Catalysts

**Figure 4:**
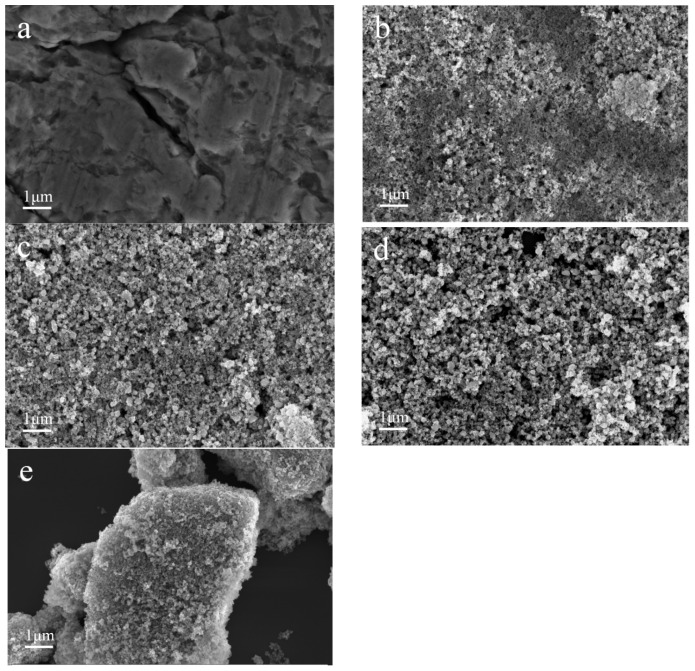
SEM images of used FeCrO*_x_* catalysts after NH_3_ decomposition reaction: (**a**) Fe, (**b**) Fe_0.75_Cr_0.25_, (**c**) Fe_0.5_Cr_0.5_, (**d**) Fe_0.25_Cr_0.75_, and (**e**) Cr.

Generally speaking, the morphologies, sizes, and phase compositions of the catalysts were changed after the ammonia decomposition reaction. The morphology of the FeCrO*_x_* catalysts after the ammonia decomposition reaction was investigated by SEM in Figure 4. The used Fe_0.75_Cr_0.25_, Fe_0.5_Cr_0.5_and Fe_0.25_Cr_0.75_ samples with higher catalytic activity exhibited similar morphologies, with small and uniform particles and secondary particle sizes that ranged from ten to one hundred nanometers, as shown in Figure 4b–d. However, for the pure Fe sample, Figure 4a displays larger-size particles exceeding nanometers, and even massive particles, which may relate to the high-temperature sintering of the catalyst during the catalytic reaction process [10,40]. Moreover, the particle size of the pure Cr sample was also relatively large, and some voids and particles were scattered on the surface of the sample after the reaction. As a result, the bimetallic and mono-metallic catalysts exhibited very different particle morphologies at the same scale (1 μm) in SEM (Figure 4). Since the SEM images reflect the morphologies of the macroscopic secondary particles, it is necessary to study the structural information on the micro scale by integrating XRD, XAFS, and other characterization methods.

**Figure 5:**
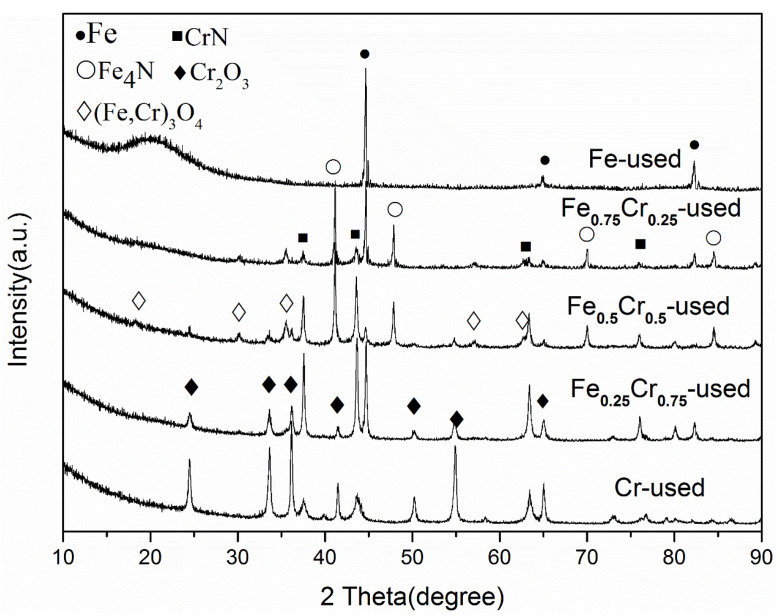
XRD patterns of used FeCrO*_x_* catalysts after NH_3_ decomposition reaction.

The XRD patterns of the used FeCrO*_x_* catalysts in Figure 5 show obvious phase transformations, as expected. For the pure iron sample (Fe_2_O_3_ before reaction), it can be observed that the diffraction peaks of Fe_2_O_3_ disappeared, and new peaks at 44° and 64°, which were attributed to the (110) and (200) planes of the cubic crystal phase of α-Fe (JCPDS No.06-0696), were detected after the ammonia decomposition test. This indicates that, during the catalytic process of the ammonia decomposition into H_2_ and N_2_, the majority of the Fe_2_O_3_ species were reduced to Fe species. In addition, such an Fe phase was found in several other Fe-containing samples, implying that this Fe phase would be the active species in this reaction. It is worth noting that, in the XRD patterns of the Fe_0.75_Cr_0.25_ and Fe_0.5_Cr_0.5_ samples that exhibited high ammonia decomposition activities, another cubic crystal phaseFe_4_N (JCPDS No.06-0627)appeared, which can be attributed to the adsorption of N atoms on the surface Fe species during the ammonia decomposition process [13,14,42,46]. This could have a significant impact on the catalytic activity of the ammonia decomposition reaction. Similarly, due to the strong adsorption and binding ability of N atoms, the phase of chromic oxide also transformed into nitride CrN (JCPDS No.65-2899) after the reaction, and with an increase in the Cr content, the cell parameters of the CrN in these samples decreased successively, indicating that the addition of Fe would cause the lattice expansion of CrN. At the same time, due to the presence of the Cr species, the Fe^3+^ species in the Fe_0.75_Cr_0.25_, Fe_0.5_Cr_0.5_, and Fe_0.25_ Cr_0.75_ sampleswere partially oxidized to Fe_3_O_4_ (JCPDS No.65-3107), rather than being entirely reduced to zero valence. Analogous to (Fe,Cr)_2_O_3_, we believe that this is also FeCr mixed oxide (Fe,Cr)_3_O_4_.

On the other hand, the XRD curve calculation for the used samples indicates that, except for a decrease in the particle size of the pure Fe sample, the other particle sizes basically increased (Table 3). The grain size of Fe in the Fe-used was 46 nm, calculated by the Scherer formula considering the characteristic peak of Fe (110), which was far different from the Fe particle size in the SEM image, further indicating that the large particles in this sample are caused by the sintering or agglomeration of Fe particles during the catalytic ammonia decomposition process. The sintering effect of iron may also play a role in its lower catalytic activity at high reaction temperatures when compared to the other FeCrO*_x_* catalysts.

From the XRD patterns, we can see that the phase composition of the FeCrO*_x_* catalyst after the reaction is relatively complex, containing more than two phases, except for the Fe-used samples. Because the diffraction peaks of these phases may be covered, their structural changes can only be recognized qualitatively based on the XRD patterns. As a result, the application of the XAFS characterization method to determine the fine structural changes in the active centers of the catalysts is urgently required.

**Figure 6:**
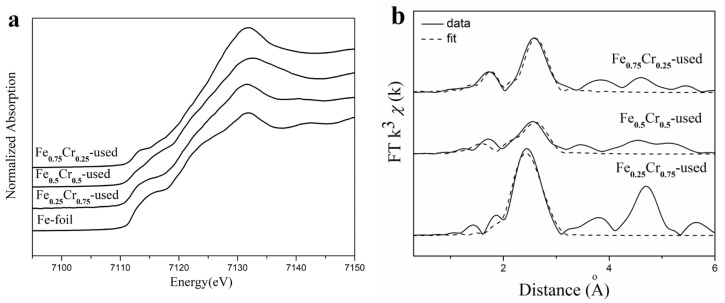
Fe K-edge XANES profiles (**a**), and EXAFS fitting results in *R* space (**b**), of used FeCrO_x_ catalysts.

The element-sensitive XAFS technique is used to determine the electronic and local coordination structures of the Fe and Cr species of the used FeCrO*_x_* catalysts. The XANES profiles of the three Fe-containing samples (Fe_0.75_Cr_0.25_-used, Fe_0.5_Cr_0.5_-used, and Fe_0.25_Cr_0.75_-used) in Figure 6a display an edge jump energy that is similar to that of the Fe-foil reference, indicating that the iron species that were presented in these samples were generally transformed into metallic states (Fe^0^) after the ammonia decomposition reaction. Since XANES is the result of the average valence of the central metal elements, we can draw a conclusion that most Fe elements exist in a zero valence state after the reaction, in the form of Fe and Fe_4_N, even though the existence of Fe^2+^/Fe^3+^ cannot be completely excluded, which is also consistent with the XRD results that were discussed previously.

For the EXAFS part, Fe/Fe_2_N/Fe_4_N models were applied to fit the extended edge region of the samples after the reaction in the R space, and the short-range local structures around the Fe atoms, such as distance (R) and coordination number (*CN*), were obtained. Figure 6b depicts the EXAFS fitting curves (dot lines) of the used samples, and the corresponding calculation results are listed in Table 4. The local structure of the Fe_0.75_Cr_0.25_ -used sample with the highest catalytic activity was similar to that of the Fe_2_N model, and the coordination number of the Fe-Fe shell at 2.74 Å was 6.5. Although both the Fe and Fe_4_N phases were detected by XRD (Figure 5), we estimate that the extended edge is a result of the multiple scattering of metal atoms, finally revealing the structure of Fe_2_N. The structure of the Fe_0.5_Cr_0.5_-used sample was more consistent with the Fe_4_N model, and the coordination numbers (*CN*) of the Fe-Fe shell were 2.7 and 5.3 at the positions of 2.55 Å and 2.70 Å, respectively. The Fe_0.25_Cr_0.75_-used sample showed very strong peaks at the positions of 2.46 Å and 2.83 Å, with corresponding coordination numbers of 6.5 and 5.1, which was more consistent with the metallic iron phase characteristics.

**Figure 7:**
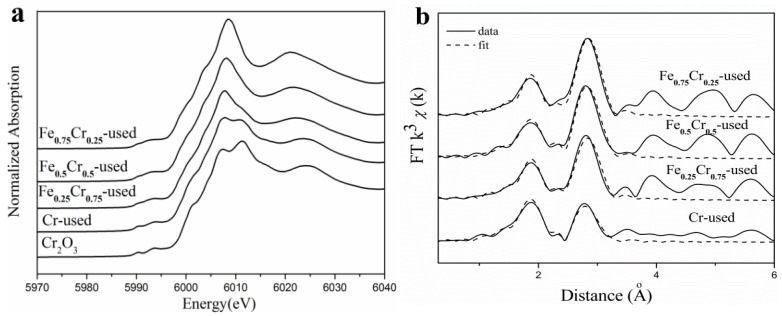
Cr K-edge XANES profiles (**a**) and EXAFS fitting results in *R* space(b) of used FeCrO*_x_* catalysts.

**Figure 8:**
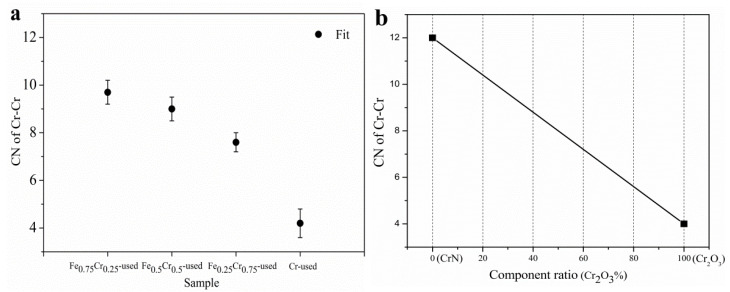
(**a**) Cr-Cr shell coordination numbers by EXAFS fitting data of used FeCrO*_x_* catalysts, and (**b**) Cr-Cr shell coordination numbers as a function of proportions between CrN and Cr_2_O_3_ model.

Furthermore, Cr K-edge (5989 eV) XAFS measurements of the used FeCrO*_x_* catalysts were also conducted, and the XANES profiles and fitted EXAFS results are shown in Figure 7 and Table 5. The edge configurations of the used FeCrO*_x_* catalysts were identical to those of Cr_2_O_3_, as guided by the XANES profiles, indicating that the Cr species were relatively stable during the reaction and were not reduced to zero valence. Since Cr existed in an oxidized Cr(III) state in the Cr_2_O_3_ or CrN models, we also needed to perform a coordination environment analysis on the EXAFS part to identify the structural changes of the sample after the reaction. A total of four Cr-containing samples were fitted in the R space, using the Cr_2_O_3_ and CrN models, respectively. The fitting curves and the data results of the two models were found to be very close, so we chose to report the results using CrN as the model, which are listed in Table 5. In the first shell, we could not distinguish the Cr-O bonds from Cr-N bonds by the bond length, R, or coordination number, *CN*, and there was no distinction between the four samples. However, for the second shell, the Cr-Cr fitting results at 2.94 ± 0.01 Å and the coordination numbers of the different samples were significantly different, that is, the coordination number of the Cr-Cr bond gradually decreased as the Cr content increased (see Table 5 and Figure 8a).

According to the standard curve of the coordination numbers, corresponding to the different phase composition ratios in Figure 8b, it can be seen that for the Cr-Cr shell, the coordination number of the pure CrN is 12, and that of the pure Cr_2_O_3_ is 4. Therefore, it was determined that the Cr species in the Cr-used (*CN* = 4.2) sample after the reaction were mainly the Cr_2_O_3_ phases, whereas the CrN phases were predominant in the Fe_0.75_Cr_0.25_-used (*CN* = 9.7) and Fe_0.5_Cr_0.5_-used (*CN* = 9.0) samples, and the CrN and Cr_2_O_3_ phases were almost evenly split in the Fe_0.25_ Cr_0.75_-used (*CN* = 7.6) sample. The XAFS analysis results are a further quantitative judgment that is based on the basic phase information that was obtained by XRD (Figure 5 and Table 3), making our analysis results more explicit and reliable.

**Figure 9:**
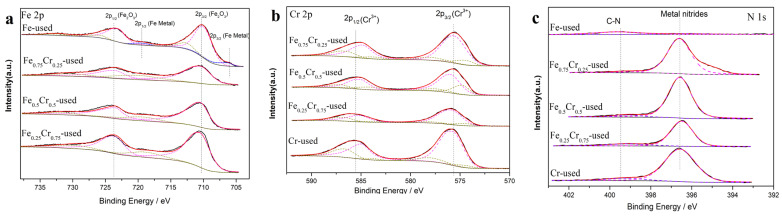
XPS spectra of used FeCrOx catalysts: (**a**) Fe 2p, (**b**) Cr 2p, and (**c**) N 1s.

X-ray photoelectron spectroscopy is used to qualitatively and quantitatively study types of elements, particularly their surface chemical valence states. The Fe2p, Cr2p, and N1s profiles of the catalysts after the reaction are shown in Figure 9. The Fe 2p subspectrum clearly shows two different peaks, Fe 2p_3/2_(Fe^3+^) at 710.6 eV and 2p_1/2_(Fe^3+^) at 723.7 eV, with an energy difference of about 13.1 eV between the two electron spin orbits, which is the structural feature of Fe_2_O_3_ [51]. At the same time, the corresponding satellite peaks, which are located near 718.9 eV and 733.3 eV, also indicate the presence of Fe^3+^ in the sample [52]. Besides the Fe_2_O_3_ signals, a new peak, corresponding to metallic Fe at about 706.1 eV, was discovered in the Fe-used sample [53]. The presence of oxidized Fe_2_O_3_ species in the used Fe sample could be ascribed to the surface Fe oxidation when the sample was exposed to air after the catalytic reaction. Then, a semi-quantitative calculation of the different components can be carried out, according to the peak areas that are occupied by different valences after the peak separation [54], but these calculations are only the results of the relative content proportion, as shown in Table 6. The Cr2p profiles of the used Cr-containing catalyst showed a similar valence and distribution of Cr in all the used catalysts, with obvious Cr^3+^ peaks [53,55], which is consistent with the XRD and XAFS results. The N 1s subspectrum is composed of two peaks (Figure 9c). Except for the Fe-used catalyst, the N1s of the other catalysts have obvious peaks at around 396.6 eV. The peak at BE 399.2eV belongs to the peak of the pyrrorole compound [56,57], and the peak at 396.6 eV belongs to the typical metal nitrides peak. Combined with the XAFS and XRD analyses, it can be concluded that the Fe_0.75_Cr_0.25_-used, Fe_0.5_Cr_0.5_-used, Fe_0.25_Cr_0.75_-used, and Cr-used samples successfully formed metal nitride species after the reaction, while the Fe-used sample did not form any related nitride species during the reaction. Again, the above shows that, after the catalyst underwent ammonia decomposition, the active components in the sample were divided into Fe and FeN_x_ species.

### 3.3. Identification of Catalytic Active Components

**Figure 10:**
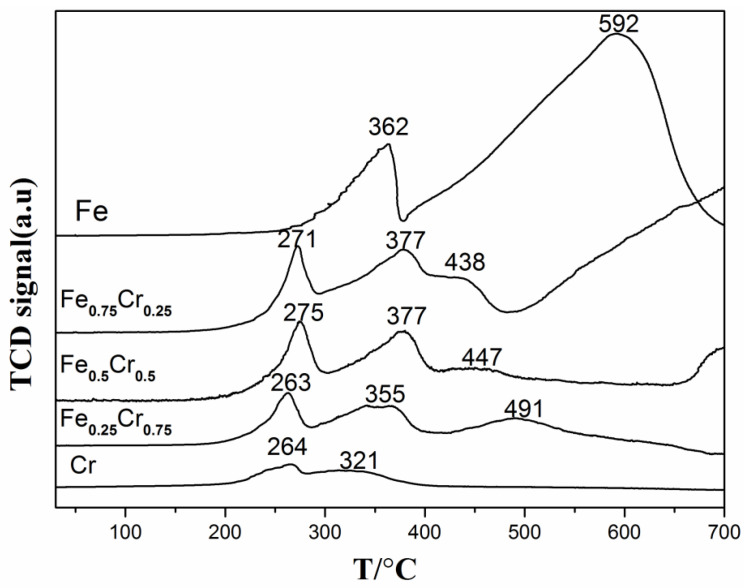
H_2_-TPR profiles of FeCrO*_x_* catalysts.

In order to further explore the structural transformations of the catalysts under a reducing atmosphere, a temperature-programmed reduction with hydrogen (H_2_-TPR) characterizations was carried out. In Figure 10, the pure Fe sample shows two reduction peaks in the hydrogen atmosphere at 362 °C and 592 °C, respectively, with the former peak corresponding to the partial reaction process of Fe_2_O_3_ to Fe_3_O_4_, and the latter peak being attributed to the total reaction process from Fe_3_O_4_ to Fe [58,59,60]. The H_2_-TPR curve of Cr_2_O_3_ shows two reduction peaks at 263 °C and 321 °C, which correspond to the reduction of the surface Cr^6+^ of the chromium oxide and the reduction of Cr^3+^ to Cr^2+^, respectively [61,62,63]. As for the reduction process of the bimetallic samples, it can be seen from Figure 10 that the first reduction peak occurs around 270 °C, due to the addition of Cr components, which can be attributed to the reduction of the surface Cr^6+^ and small particles of Fe_2_O_3_ due in part to doping of Cr, while the second reduction peak at 350–370 °C should be ascribed to the combined reduction of Fe_2_O_3_ to Fe_3_O_4_ and a Cr^3+^ partial reduction, and the third peak should be attributed to the reduction of Fe_2_O_3_ to Fe_3_O_4_, and a partial reduction of Fe_3_O_4_ (before 500 °C). In addition, we also compared the corresponding reaction temperatures of the different samples at the H_2_-TPR reduction temperature (*T*_R_) and 50% NH_3_ conversion rate (*T*_50_) in Table 7. The reaction temperature at the 50% NH_3_ conversion was discovered to be after the third reduction peak, meaning that the state of Fe was tending to zero, which is also consistent with our previous conclusion.

In particular, in order to investigate the FeCr catalyst’s active species in the ammonia decomposition reaction, it is necessary to understand the causes for the deactivation of Fe-based catalysts: (1) the sintering effect of Fe nanoparticles under high-temperature reaction conditions; and (2) Fe-based catalysts are easily nitrided in an atmosphere with a high ammonia concentration and undergo a phase transition to generate the corresponding metal nitrides, but it has been debatable up until now whether the latter can lead to a catalyst deactivation. A series of findings from Arabczvk’s research group [11,12,64,65] showed that: (1) the nitriding degree increased along with the growth of the Fe particle size; and (2) when the chemical composition of the nitriding substance became α-Fe (N), the ammonia decomposition activity increased with the increase of the nitriding degree. However, the ammonia decomposition activity of the material was reduced with a rise in the Fe nitridation degree when the nitridated granules were composed of mixed α-Fe(N) and γ′-Fe_4_N phases. When Fe_2_O_3_/CMK-5 was used as the ammonia decomposition catalyst by Lu’s research group [45], three crystal phases of γ-Fe_2_O_3_, Fe_4_N, and Fe_3_N_1.233_, with particle sizes larger than that of Fe_2_O_3_ in mesoporous carbon, were found on the surface of the catalyst after the reaction at 700 °C. The deactivation of the Fe_2_O_3_/CMK-3 catalyst was thought to be caused primarily by the formation of ferric nitride species. It is worth noting that Guo’s group [42] investigated the ammonia decomposition activity of an Fe catalyst by using a dielectric barrier discharge method, and found that although the Fe_2_N catalyst showed some ammonia decomposition activity, they still believed that the metallic Fe was the real active phase, which was easy to form less active Fe_4_N with the N_ad_ atoms that were adsorbed on the surface, and to cause poisoning and deactivation [12]. The reason for this is that, before the XRD analysis, the catalyst after the reaction, by shutting off the plasma, was still in the NH_3_ flow until it was completely cooled, while the ammonia decomposition took place during this period. Therefore, part of the surface metallic Fe species eventually transformed into Fe_3_N/Fe_4_N nitrides by the firmly adsorbed N atoms.

Although nitriding is thought to be a major cause for the deactivation of Fe-based catalysts, some researchers believe that Fe nitrides are the active species, rather than the deactivated species. Ohtsuka’s group [13,66] investigated the reaction mechanism of an ammonia decomposition that was catalyzed with Fe, by in situ XRD and NH_3_-TPD-MS techniques. They discovered that Fe first reacted with NH_3_ to form Fe_4_N, Fe_3_N, and H_2_, and that once the reaction temperature reached 350 °C, these nitrides decomposed to form Fe and N_2_, which were just intermediate products in the ammonia decomposition process. Su’ group [46] investigated the activity and stability of the residual Fe in CNTs as a catalyst for ammonia decomposition, and found that neither Fe nor Fe carbides were the active components of the catalyst, but that the FeN*_x_* that was generated after the reaction could be the active component for this reaction. Schuth’s research team [44] studied the phase transitions of Alpha-Fe_2_O_3_@SiO_2_ catalysts, using in situ XRD measurements at various temperatures in an ammonia atmosphere. As a result, the catalyst mainly existed in the form of ferric oxide and FeN*_x_* below 650 °C, and transformed to Fe and a trace of FeN*_x_* above 650 °C.

Through the means of characterizing FeCrO*_x_* bimetallic catalysts before and after the ammonia decomposition reaction, especially in combination with the phase information from the XRD patterns and the fitting results of the XAFS, we can draw a conclusion that the metallic Fe and FeN*_x_* species would be the active components of our FeCrO*_x_* catalysts within the ammonia decomposition reaction. Figure 3 shows that all four Fe-based samples (Fe, Fe_0.75_Cr_0.25_, Fe_0.5_Cr_0.5_, and Fe_0.25_Cr_0.75_) exhibited relatively high catalytic activities. According to the XRD patterns, the Fe-used and Fe_0.25_Cr_0.75_-used catalysts simply showed diffraction peaks of the α-Fe phase (without considering the phase containing Cr), and the fitting curve of the latter was also the pure Fe (Figure 5, Table 4), implying that the metallic Fe species should be the active phase of the catalysts. Moreover, the phases of the Fe_0.75_Cr_0.25_-used and Fe_0.5_Cr_0.5_-used samples were quite complicated, with a large number of FeN*_x_* phases detected and confirmed by the corresponding EXAFS fitting results (Figure 7, Table 4). Therefore, FeN*_x_* species have been identified as the active species in the ammonia decomposition reaction. In addition, by comparing the characterization results of the Cr-containing and Cr-free samples, we can also conclude that Cr effectively inhibits the high-temperature sintering process of Fe catalysts, and that the generated CrN phase is relatively stable after the reaction.

## 4. Conclusions

In this study, a series of FeCrO*_x_* bimetallic oxide nanocatalysts with varying metal ratios were created via a urea two-step hydrolysis approach. The as-synthesized catalysts demonstrated a good catalytic performance for the ammonia decomposition reaction, with the Fe_0.75_Cr_0.25_ catalyst exhibiting the highest ammonia decomposition activity at 650 °C and a complete conversion of the ammonia. The structure–activity relationship of the FeCr metal oxide catalyst in the catalytic ammonia decomposition was further investigated using the SEM, XRD, XPS, and XAFS techniques. We believe that both the metallic Fe and FeN*_x_* species are the active components of our FeCrO*_x_* catalysts in the ammonia decomposition reaction. The bimetal samples exhibited better catalytic activity than the mono-metal samples, and Fe_0.75_Cr_0.25_ was the ideal ratio among a number of the bimetal catalysts. While not losing too much of the active site for the ammonia decomposition reaction, the doping of a modest quantity of Cr can effectively prevent the high-temperature sintering of Fe. It is believed that the series of research findings from this work will have significant guiding implications for the design and development of efficient and stable ammonia decomposition catalysts in the future, and will also aid in better understanding other heterogeneous catalytic processes.

## Figures and Tables

**Table 1:** Physical properties of fresh FeCrO*_x_* catalysts.

Sample	Surface Atomic Ratio (Fe/Cr) *^a^*	Lattice Constants (Å) *^b^*	*D* (nm) *^c^*	S_BET_ (m^2^/g) *^d^* Fresh/Uesd
**Fe**	100/0	*a* = 5.0329(1) *c* = 13.7462(2)	56	13/-
**Fe_0.75_Cr_0.25_**	67.2/32.8	*a* = 5.0192(4) *c* = 13.708(1)	25	62/34
**Fe_0.5_Cr_0.5_**	45.0/55.0	*a* = 4.9863(3) *c* = 13.6357(7)	16	84/34
**Fe_0.25_Cr_0.75_**	30.2/69.8	*a* = 4.9607(6) *c* = 13.587(1)	16	51/23
**Cr**	0/100	*a* = 4.9393(1) *c* =13.5549(1)	27	39/17

*^a^* Determined by XPS; *^b^* calculated from the XRD patterns of (Fe,Cr)_2_O_3_; *^c^* calculated from Scherrer formula at the (1 0 4) crystal plane of (Fe,Cr)_2_O_3_; and *^d^* calculated from N_2_ adsorption points in the relative pressure range between 0.06 and 0.30.

**Table 3:** Phase composition, lattice constants (Å), and crystal grain size (*D_XRD_*) of used FeCrO*_x_* catalysts.

Sample	Phase Composition	(Fe,Cr)_2_O_3_	(Fe,Cr)_3_O_4_	Fe	Fe_4_N	CrN
**Fe-used**	lattice constants *a*(Å) *^a^* and crystal grain size *D* (nm) *^b^*	-	-	2.8667(1) *D* = 46	-	-
**Fe_0.75_Cr_0.25_-used**		-	8.3778(1)	2.8667(1) *D* = 53	3.7967(1) *D* = 47	4.1476(1)
**Fe_0.5_Cr_0.5_-used**		*a* = 4.9671(3) *c*=13.5875(7)	8.3776(2)	2.8670(1)	3.7984(1) *D* = 49	4.1469(2) *D* = 37
**Fe_0.25_ Cr_0.75_-used**		*a* = 4.9622(2) *c*=13.5762(5) *D* = 29	-	2.8668(2) *D* = 47	-	4.1449(1) *D* = 38
**Cr-used**		*a* =4.9638(1) *c*=13.5581(4) *D* = 32	-	-	-	4.1438(1) *D* = 38

*^a^* Calculated from the XRD patterns; and *^b^* calculated from Scherrer formula at the related phase.

**Table 4:** Fe K edge EXAFS fitting results of used FeCrO*_x_* catalysts *^a^*.

Sample	Fe-N		Fe-Fe		D. W.	∆*E*_0_ (eV)
	*R* (Å)	*CN*	*R* (Å)	*CN*		
**Fe_0.75_Cr_0.25_-used ^α^**	1.94 ± 0.01	2.8 ± 1.1	2.74 ± 0.03	6.5 ± 1.1	0.006(Fe) 0.003(N)	9.5 ± 3.2
**Fe_0.5_Cr_0.5_-used ^β^**	1.71 ± 0.03	−0.9 ± 0.6	2.55 ± 0.03 2.70 ± 0.01	2.7 ± 1.7 5.3 ± 1.9		7.6 ± 6.2
**Fe_0.25_Cr_0.75_-used ^γ^**	_	_	2.46 ± 0.01 2.83 ± 0.01	6.5 ± 0.8 5.1 ± 1.3		2.9 ± 3.8

^a^*R*: distance; *CN*: coordination number; *D.W.*: Debye–Waller factor; and ∆*E*_0_: inner potential correction to account for the difference in the inner potential between the sample and the reference. ^α^ The fitting results were modeled by Fe_2_N. ^β^ The fitting results were modeled by Fe_4_N. ^γ^ The fitting results were modeled by Fe.

**Table 5:** Cr K-edge EXAFS fitting results of used FeCrO*_x_* catalysts* ^a^*.

Sample	Cr-N/O		Cr-Cr		D. W.	∆*E*_0_ (eV)
	*R* (Å)	*CN*	*R* (Å)	*CN*		
**Fe_0.75_Cr_0.25_-used**	2.02 ± 0.04	6.4 ± 0.6	2.95 ± 0.02	9.7 ± 0.5	0.005(Cr)	9.9 ± 0.5
**Fe_0.5_Cr_0.5_-used**	2.02 ± 0.05	6.3 ± 0.5	2.94 ± 0.01	9.0 ± 0.5		
**Fe_0.25_Cr_0.75_-used**	2.02 ± 0.05	6.2 ± 0.5	2.94 ± 0.01	7.6 ± 0.4		
**Cr-used**	2.00 ± 0.07	6.6 ± 0.6	2.93 ± 0.00	4.2 ± 0.6		

*^a^ R*: distance; *CN*: coordination number; *D.W.*: Debye–Waller factor; and ∆*E*_0_: inner potential correction to account for the difference in the inner potential between the sample and the reference.

**Table 6:** XPS fitting results for used FeCrOx catalysts.

Sample	Chemical State				
	Fe_2_O_3_	Fe Metal	Cr^3+^	Metal Nitrides	C-N
	(%)	(%)	(%)	(%)	(%)
**Fe-used**	89.86	10.14	-	-	-
**Fe_0.75_Cr_0.25_-used**	100	0	100	97.22	2.78
**Fe_0.5_Cr_0.5_-used**	100	0	100	94.39	5.61
**Fe_0.25_Cr_0.75_-used**	100	0	100	85.38	14.62
**Cr-used**	-	-	100	92.42	7.58

**Table 7:** H_2_-TPR reduction temperatures (*T*_R_) and temperatures at 50% NH_3_ conversion (*T*_50_) of FeCrO*_x_* catalysts.

Sample	*T*_R_ (°C) *^a^*	*T*_50_ (°C) *^b^*	H_2_ Consumption (μmol/g) *^c^*
**Fe**	362, 592	532	21,328
**Fe_0.75_Cr_0.25_**	271, 377, 438	490	7498
**Fe_0.5_Cr_0.5_**	275, 377, 447	513	6552
**Fe_0.25_Cr_0.75_**	263, 355, 491	520	4241
**Cr**	264, 321	608	1563

*^a^* Data are from the H_2_-TPR curves in Figure 10; and *^b^* data are from the ammonia decomposition activity curves in Figure 3. *^c^* Calculated from the H_2_-TPR curves.

## Data Availability

Not applicable.
